# Delirium Diagnostic and Screening Instruments in the Emergency Department: An Up-to-Date Systematic Review

**DOI:** 10.3390/geriatrics1030022

**Published:** 2016-09-01

**Authors:** José Mariz, Teresa Costa Castanho, Jorge Teixeira, Nuno Sousa, Nadine Correia Santos

**Affiliations:** 1 Life and Health Sciences Research Institute (ICVS), School of Health Sciences, University of Minho, Campus Gualtar, 4710-057 Braga, Portugal; josemariz@ecsaude.uminho.pt (J.M.); id4515@alunos.uminho.pt (T.C.C.); njcsousa@ecsaude.uminho.pt (N.S.); 2 ICVS/3B’s—PT Government Associate Laboratory, 4710-057 Braga/Guimarães, Portugal; 3 Emergency Department, Hospital de Braga, 4710-243 Braga, Portugal; diretor.servico.urgencia@hospitaldebraga.com.pt; 4 Clinical Academic Center—Braga, 4710-243 Braga, Portugal

**Keywords:** delirium, emergency department, CAM-ICU, organic brain syndrome, RASS

## Abstract

Background: Emergency care systems are at the core of modern healthcare and are the “point-of-entry/admission” into the hospital for many older/elderly patients. Among these, it is estimated that 15% to 30% will have delirium on admission and that over 50% will develop it during their stay. However, appropriate delirium diagnostic and screening still remains a critical area of need. The goal of this review is to update the field, exploring target areas in screening methods for delirium in the Emergency Department (ED), and/or acute care units, in the older population. Methods: A systematic review was conducted to search screening/diagnostic methods for delirium in the ED and/or acute care units within the ED. Results: Seven different scales were identified. Of the identified instruments, the Confusion Assessment Method (CAM) for the Intense Care Unit (CAM-ICU) was the most widely used. Of note, a brief two-step approach for delirium surveillance was defined with the Delirium Triage Screen (DTS) and the Brief Confusion Assessment Method (bCAM), and the diagnostic accuracy of the Richmond Agitation-Sedation Scale (RASS) for delirium had a good sensitivity and specificity in older patients. Conclusion: The CAM-ICU appears as the potential reference standard for use in the ED, but research in a global approach of evaluation of actual and past cognitive changes is still warranted.

## 1. Overview

Emergency care systems are at the core of modern healthcare. Their primary role is to provide high-quality care to patients regardless of when they need medical help or what they present with [1]. In the Emergency Department (ED) (and/or intermediate or acute care units within these), much of the problem regarding delirium incidence and prevalence relates, in great part, to the unique environment: intense time demands on providers and high volume of patients. Together, these aspects can both: (i) render the caring for older adults challenging; and (ii) hinder the use and validation of screening tools. This occurs despite any positive impact that this latter work would in itself yield, including early identification of delirium and translation into appropriate treatment measures. Regarding delirium, the problem is compounded when considering that the older/elderly population uses emergency care facilities more often than younger individuals [2] and age is one of the principal risk factors for delirium onset. Studies of delirium prevalence have indicated that 15% to 30% of the older/elderly population will have delirium on admission to hospital and over 50% will develop it during their stay (for example, [3,4]). These data are collaborated by a recent and very relevant review by Inouye et al. (2014) indicating that delirium is present in 8%–17% of all elderly people and 40% of nursing home residents, with the authors also noting that presence of delirium in the community is rather low (1%–2%) but onset is conducive to an ED admission. Delirium associates with relevant indicators such as increased mortality, including after discharge from the hospital, and its presence in the emergency department (ED) is a predictor of a longer hospital stay [3,4,5].

Still, despite the findings, there has been surprisingly a lack of studies in the ED context. For instance, to our knowledge, to date, there are no studies on delirium incidence in the ED amongst older/elderly individuals. This is also particularly true concerning physician detection rates and efficacious detection methods, such that the detection of delirium in the ED continues to be indicated as a “high yield” research objective [4]. It is, furthermore, also identified as a quality indicator of geriatric assessment in the ED [6]. Stemming from this need, LaMantia et al. (2014) previously published a relevant systematic review on screening tools of delirium in the ED. Since then, there have been other significant advances which meet what the authors had indicated as necessary targets in order to move the field of delirium identification and management forward within the ED [6]. Chiefly amongst these advances: (i) Han and colleagues validated the Confusion Assessment Method for the Intense Care Unit (CAM-ICU) for the ED [7], continuing work on brief tools for delirium in this setting, which responds to the need for more rapid assessments of acute brain function; and (ii) a novel screening approach was tested using the Richmond Agitation-Sedation Scale (RASS), a brief attention test followed by an adaptation of the CAM-ICU [8,9].

Here, providing a further update to the advances in the field, the goal of this review is to explore target areas in screening methods for delirium in the ED and/or acute care units, with focused attention on rapid screening tools adapted to these settings. More so, we seek to inform in terms of time consumed administering the available instruments, which is one of the more important challenges to do research in delirium in the ED. The use of psychometric characteristics to better guide the emergency care provider in choosing the screening tool, and practical cues on the application of the screening tools to help the clinician better choose depending on emergency load, are also considered.

## 2. Literature Review

A systematic search of the literature was conducted in the Science Direct and MEDLINE/PubMed databases using a combination of MeSH terms “organic brain syndrome”, “delirium”, “acute confusional state”, “emergency” and “emergency department”. The databases were searched from their inception to November 2015 for relevant articles published in English, French, Spanish and Portuguese. The term “organic brain syndrome” was included to consider previous literature when the term was commonly used in referring to delirium. Two separate researchers (J.M., an emergency physician, and T.C.C., a psychologist) conducted independently the search. The generated lists were compared to obtain a compiled list of potential studies for inclusion. The PICOT framework and the search strategy of LaMantia et al. (2014) [6] were considered.

Only prospective studies of patients aged 65 years of age and older were considered, and only if the characteristics of the used (screening/diagnostic) instruments were described. Out-of-hospital evaluations and interventional studies (on delirium) were excluded from the review, as were commentaries and/or author opinions on neuropsychological (delirium) instruments. Acute patient units and/or Intermediate Units were only considered if integrated and/or physically located (with)in the ED, given that in great part these are found to function with the same staff and constraints of the ED. Of note, the definition of Acute Care Unit is not uniform across continents and countries, including varying in what kind of patients are admitted and when. If used, the Diagnostic and Statistical Manual of Mental Disorders (DSM) was not considered a screening instrument in itself, but rather the gold standard criteria (including if applied by non-psychiatrists). All tools used to assess delirium (both diagnostic and screening) were considered in the analysis; in the final quantitative step, all tools were supported by validation studies.

**Figure 1:**
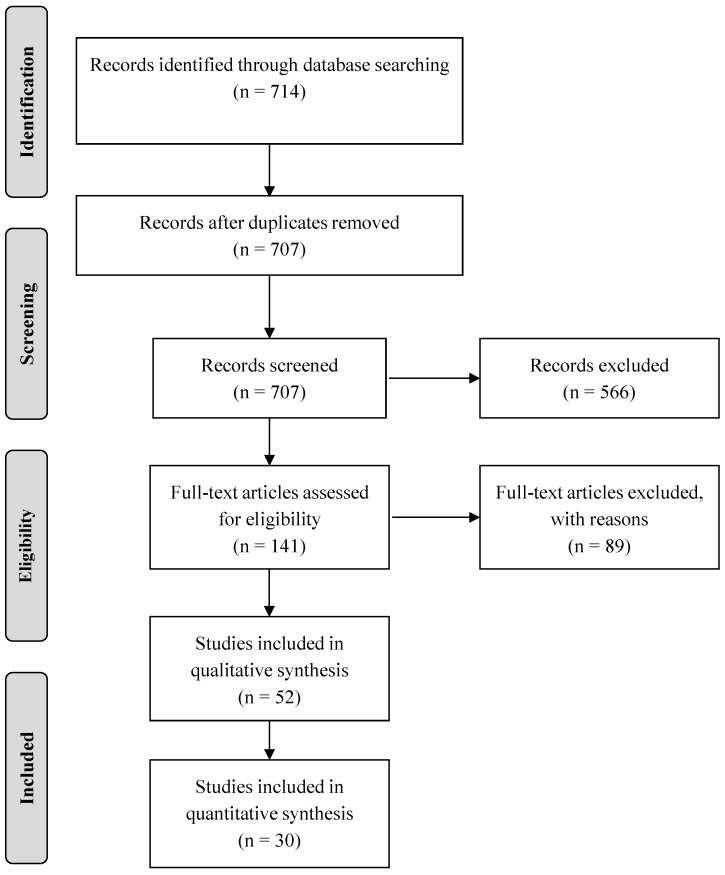
Flow diagram of the literature review.

Summarily, a total of 714 citation titles were identified in the search. Of these, 707 potentially relevant abstracts were screened, of which 141 articles were selected for detailed analysis (i.e., met the exclusion and inclusion criteria). Of the full articles read and assessed for eligibility, a final 30 were included in the systematic review. From the analysis, a total of seven delirium screening tools were identified as being used in the ED. The search procedure is summarized in Figure 1, following the “Preferred Reporting Items for Systematic Reviews and Meta-Analyses (PRISMA)” flow diagram template. Table 1 summarizes the instruments, including information on author (date of publication), population, type of study, setting (country), purpose and main findings of the work. Table 2 summarizes the (original) author, validation sample/study, administration time, sensitivity and specificity measures, and additional validation studies in other countries, of the delirium screening instruments here identified.

## 3. Delirium Screening Instruments in the ED and/or in Acute Care Units

A total of seven different instruments were identified for delirium screening/diagnostic: Confusion Assessment Method (CAM); modified Confusion Assessment Method for the Emergency Department (mCAM-ED); Confusion Assessment Method for the Intensive Care Unit (CAM-ICU); Delirium Triage Screen (DTS); Brief Confusion Assessment Method (bCAM); Neelon and Champagne Confusion Scale (NEECHAM); and the Richmond Agitation Sedation Scale (RAAS). The latter was considered in the analysis; albeit being a sedation scale not directed for delirium assessment, there are enough data to consider it a screening scale with a good psychometric performance [36]. The DTS and the bCAM were considered as separate instruments, although it is proposed as a two-step approach in the ED environment [30].

### 3.1. Confusion Assessment Method (CAM)

The CAM [8] is overall considered the standardized, structured instrument to identify patients with delirium. It utilizes observation and interaction with the patient to provide data to which a simple algorithm can be applied to determine if delirium is present or not. Specifically, the CAM includes two parts. Part one screens for overall cognitive impairment and part two considers the following features: (1) acute onset of mental status changes or a fluctuating course; (2) inattention; (3) disorganized thinking; and (4) altered level of consciousness. A positive diagnosis of delirium requires the presence of Features (1) and (2), and either Feature (3) or (4). The instrument was validated for use by geriatricians and it takes approximately 5 min to fill out. Using psychiatric assessment as the gold standard, the CAM has shown a sensitivity of 94% to 100%, a specificity of 90% to 95% for the detection of delirium and a high inter-observer reliability [8]. This tool is translated and validated for use in various non-English speaking populations. Despite the CAM’s possibility to identify the presence or absence of delirium in a quick and easy way, it does not assess the severity of the condition.

### 3.2. Modified Confusion Assessment Method for the Emergency Department (mCAM-ED)

The mCAM-ED is an algorithm developed by Grossman et al. (2014) [22] for delirium screening, detection and management in older ED patients. An integral part of the algorithm is the modified Confusion Assessment Method for the Emergency Department, a feasible approach based on the original short version of the CAM. The scale requires a maximum of one minute to evaluate attention and 3 to 5 min to complete the whole instrument. The sensitivity and specificity reported in the original study was only for the informal delirium assessment. The authors concluded that the study indicated for the need for a standardized, formal delirium screening and assessment instrument in the ED [22].

### 3.3. Confusion Assessment Method for the Intensive Care Unit (CAM-ICU)

The CAM-ICU [7] is a modified version of the CAM for use in the ICU and/or in critically ill patients. Overall, it considers the same CAM features of delirium evaluation. The main difference between the scales is that the CAM requires clinical judgment to assess for all four features, while the CAM-ICU encompasses brief neuropsychiatric assessments to determine inattention (Feature (2)) and disorganized thinking (Feature (3)). Additionally, the CAM-ICU is easier and faster to apply (less than 2 min to complete), making it ideal for the busy ED environment and attending to the critical status of the patient [8]. The sensitivities were 72.0% and 68.0% in the Emergency physicians and Research assistants, respectively. The CAM-ICU’s specificity was 98.6% [27]. However, as a drawback, while the CAM-ICU attempts to evaluate disturbances in attention and cognition, it fails to capture their fluctuating nature since the evaluation is done at one point in time.

### 3.4. Delirium Triage Screen (DTS)

The DTS [30] was designed for application in a very brief length of time (less than 20 s) so that it could be easily integrated into the clinical environment. It consists of two components: level of consciousness and attention. As the DTS has a sensitivity of 98%, it is considered that delirium can be ruled out in this case with no need for additional delirium testing. Nevertheless, since the DTS is 55% specific, other confirmatory evaluations are warranted (specifically using the CAM or bCAM) to rule in delirium [30]. Of note, the bCAM (as well as the DTS) are part of the Geriatric Emergency Department Guidelines.

### 3.5. Brief Confusion Assessment Method (bCAM)

The bCAM [31] is an adaptation from the full CAM and it comprises four questions that have been widely validated to make diagnosis of delirium: two fundamental features (acute onset and fluctuating course, and inattention), and two secondary features. The bCAM takes less than 2 min to perform and it was designed to improve CAM sensitivity and enhance its brevity [30] with the addition of an inattention task in which it is required to the patient to recite the months backwards from December to July.

### 3.6. Neelon and Champagne Confusion Scale (NEECHAM)

The NEECHAM Confusion Scale [32] is an observational scale with nine interactive items, divided in three subscales: processing, behavior and physiological control. A score of 30 indicates normal function and 0 severe confusion, with a range of 25–26 as “at risk for confusion”. It was developed for use as a rapid bedside assessment by nurses and has been applied on several samples and settings to detect delirium in the early stages, follow-up on progress and identify regression of a confusional state [37,38]. This instrument shows sensitivity and specificity values ranging from 30% to 95% and 78% to 92%, respectively. An additional advantage of the NEECHAM is its sensitivity to both the hyperactive and hypoactive variants of delirium [39].

### 3.7. Richmond Agitation Sedation Scale (RASS)

The RASS [34] is a measure of the level of arousal, being commonly used in clinical practice and research setting to assess for depth of sedation. Nonetheless, it has been incorporated into several delirium assessments, such as the bCAM and the CAM-ICU, to assess for level of consciousness [36]. It has a 10 level scale ranging from +4 (“combative) to −5 (“unrousable”) that can be briefly rated. The RASS takes less than one minute to complete and can be assessed by simply observing the patient during routine interactions and does not require additional cognitive testing [10]. This tool has been demonstrated to have excellent inter rater reliability and excellent validity when compared, for instance, to other sedation scales [40]. The diagnostic accuracy of the RASS for delirium in older patients, when applied by a physician, had a sensitivity of 82% (71.4%–92.6%) and a specificity of 85.1% (81.4%–88.8%) for a RASS other than 0, and for a RASS > +1 or < −1 had a sensitivity of 16.0% (5.8%–25.2%) and a specificity of 99.7% (99.2%–100%). Similar results were obtained when the RASS was applied by the research assistant [10].

## 4. Concluding Remarks

This review provides an update on delirium diagnostic and screening tools for use in the ED. Here, seven different scales were identified for evaluation/assessment of delirium in the ED setting. Comparing with previous reviews of literature [6] some important differences should be noted, namely that prior work identified a different group of scales. Of these, four overlap with the ones here comprised: CAM, CAM-ICU, CAM-ED, and NEECHAM. Here, we further identified the mCAM-ED, DTS, bCAM and RASS, while the previous authors consider the Diagnostic and Statistical Manual criteria (DSM) and the Delirium Rating Scale (DRS) in their analysis. Other differences and/or considerations are finally also warranted. First, here we do not consider that the original study by Lewis et al. (1995) [9] deems a formal change of designation to CAM-ED. Second, the recent work of Grossman et al. (2014) [22] has been included, which formally addresses the question of the CAM adaptation for the ED (termed mCAM-ED); thus, considering it its own independent instrument, although the study did not have sufficient power to calculate sensitivity and specificity. Finally, of note, here the OBS scale was not included if considering that the studies reporting on it were not conducted in the ED.

It is relevant to note the advances in the field since 2014. A total of three new delirium screening scales for the ED are indicated: DTS, bCAM and mCAM-ED, if considering the DTS as the bCAM as separate instruments. In addition, an important breakthrough in the field is of particular note with the formal validation of the CAM-ICU for the ED. The CAM-ICU is highly specific in older ED patients, regardless of administration by research assistants or emergency physicians [7]. However, this is true at the cost of a moderate sensitivity. The CAM-ICU authors addressed this issue in two separate but related studies [7,36]. On a first approach, the importance of a brief and easy-to-use delirium assessment tool appropriate for the fast-paced ED environment was considered with the validation of the CAM-ICU; and, within this up-to-date review, it is important to note that only the study of Sufolletto et al. (2013) did address this topic [27]. A brief and novel two-step approach for delirium surveillance was later defined with the DTS and the bCAM. A negative DTS essentially rules-out delirium and reduces the number of formal delirium assessments needed, increasing screening efficiency; thereafter, following with the bCAM, a balance between brevity and diagnostic accuracy is achieved, and it is an effective rule-in delirium assessment. Third, we included in this review the RASS scale. Although this instrument is not a delirium screening tool per se, the RASS scale has been demonstrated to have a very good specificity for detecting delirium in patients older than 65 years old, with a cut-off >+1 or <−1. The scale may be a reasonable alternative to monitor for delirium in the ED, especially when ED health care providers are faced with significant time constraints [36].

As highlighted in other general reviews about delirium scales [41], only the instruments which are used for diagnosis (i.e., CAM and CAM-ICU) and that are based on various Diagnostic and Statistical Manual criteria, have good to excellent reliability and fair to good validity. In this sense, only the studies of Han and colleagues [4,7,30,36] address the main psychometric characteristics of delirium in the ED, based on the CAM attributes, and explored its sensitivity and specificity. From this frame, it is here proposed that delirium research should evolve to focus in novel attention tests and testing their performance against CAM-ICU and DTS/bCAM approach(es), in order to be feasible, useful and of needed sensitivity/specificity in the particular setting of the fast-paced emergency care units.

Another problem that should be addressed and clarified in the future is the distinction between the acute setting of delirium, secondary to medical illness, and the (expected) cognitive decline in ageing, irrespective of the underlying medical condition. That is, the current tools may not properly distinguish between an acute cognitive “failure” (delirium, which is considered to be “organic”) from one that is sustained (underlined by reasons other than delirium, in which ageing itself plays a relevant role), albeit both may co-exist.

Cognitive disturbances are a “part and/or parcel” of delirium, and thus cannot be disregarded [41]. Perhaps due to this, and also because the onset itself of delirium and that which triggers it is not completely yet clarified, many of the instruments which have been primarily designed to assess disturbances in cognitive functions have been also been used for screening for delirium [41]. Nonetheless, as pointed by Grover et al. (2012) [41], particularly some of the earlier instruments which were used to assess the severity and phenomenology of delirium did not assess the cognitive functions comprehensively; that is, these are useful as supplementary tools for assessment of cognitive functions in patients of delirium. These include the MMSE [42], Cognitive test for Delirium (CTD) [43], Clock Drawing Test [44], Digit Span Test [45], Vigilance “A” test [44], Mental State Questionnaire (MSQ) [46,47] and the Short Portable Mental Status Questionnaire [48]. Here it is argued that delirium diagnosis should comprise a two-step approach: (i) cognitive screening with a rapid assessment tool, which should be overall acceptable for different populations and literacy levels; and (ii) delirium diagnosis/confirmation with a delirium tool. Delirium is more common in the elderly, some of whom also suffer from dementia; hence, it is also important to have an understanding of baseline cognitive functions of patients before considering the cognitive disturbances as part of the delirium. In fact, differentiating dementia from delirium is a challenge for health professionals. Among older/elder individuals, up to two-thirds of cases of delirium are superimposed with dementia, and delirium may lead to severe cognitive impairment, and many patients who have experienced delirium are at greater risk to develop dementia [23,49]. More so, patients with dementia present characteristics very similar to the symptoms of delirium profile [50]. On this, besides cognitive scales that can help to discriminate these conditions in the moment of screening and diagnosis, electroencepholography (EEG) already showed a great specificity and adequate sensitivity [51]. Caregivers’ information or informant-based screening tools are also crucial to determine if there has been an acute decline (a feature of delirium), but also to verify if there has also been a much more prolonged decline in time (characteristic of dementia). This kind of instruments/information, such as the Informant Questionnaire on Cognitive Decline in the Elderly (IQCODE; [52]) can be less likely to be influenced by the acute condition of the patient and can complement the other performance tools when used in combination.

In conclusion, besides a wide consensus on the importance of recognizing delirium in the ED, there are still few (albeit strong) attempts to clarify this important issue. Among the instruments considered, the CAM-ICU is the most widely used, although its validation is in single center studies. Despite its limitations, it surfaces as the most favorable instrument for use in critical care because of its accuracy, brevity, and ease of use by clinical and lay interviewers. In the future, research in a global approach of evaluation of actual and past cognitive changes should be considered and validation of these delirium assessments in other centers will be critical to determine which delirium assessment would be the most accurate for the ED settings. The balance between a comprehensive evaluation and the necessary brevity of the screening must, however, remain in the forefront.

## Figures and Tables

**Table 1:** Summary of studies using delirium screening tests.

Author (Date of Publication)	Population	Type of Study	Setting (Country)	Purposes
Confusion Assessment Method (CAM) (Inouye et al. 1990) [8]				
Lewis et al. (1995) [9]	385 elderly patients	Cohort study	Emergency department (Canada)	Determine the likely presence of delirium and to examine the sensitivity of emergency physician’s routine histories and physical evaluations to identify this disorder.
Élie et al. (2000) [10]	447 elderly patients	Comparative study	Primary acute care University-affiliated hospital (Canada)	Determine the prevalence of delirium in emergency department and the sensitivity and specificity of a conventional assessment by a physician for the detection of delirium.
Fabbri et al. (2001) [11]	100 elderly patients	Validation study	Emergency Room of a teaching hospital (Brazil)	Investigate CAM’s reliability of its Brazilian version as well as its validity.
Monette et al. (2001) [12]	116 elderly patients	Prospective study	Emergency Room (Canada)	Compare the results of the CAM assessment obtained by a trained non-physician interviewer and those obtained by a geriatrician.
Hustey et al. (2002) [13]	297 elderly patients	Prospective observational study	Urban teaching hospital emergency department (United States)	Determine the prevalence of mental status impairment in elderly emergency department and to assess documentation of and referrals by emergency physicians for mental status impairment after discharge
Hustey et al. (2003) [14]	271 elderly patients Family members and other people close to the participant were also interviewed regarding CAM to account for the fluctuating nature of delirium	Prospective cross-sectional study	Urban teaching hospital emergency department (United States)	Determine the effect of screening evaluations for mental status impairment and prospectively assess recognition of mental status by emergency physicians.
Kakuma et al. (2003) [15]	30 delirious and 77 nondelirious older individuals Family members and other people close to the participant were also interviewed regarding other measures to account for premorbid cognitive status and functional abilities.	Prospective study with 18 months of follow-up	Emergency department (Canada)	Determine if prevalent delirium is an independent predictor of mortality in older patients seen in the emergency department and discharged home without admission.
Naughton et al. (2005) [16]	110 elderly patients	Pretest and posttest study	University-affiliated hospital (United States)	Admit cognitively impaired and older individuals with delirium from the emergency department to an acute geriatric unit and improve outcomes for cognitively impaired and delirious older adults
Vida et al. (2006) [17]	259 elderly patients	Prospective cohort study	Emergency Department of University teaching and General Hospitals (Canada)	Determine if patients with delirium show poorer ADL, BADL or IADL at 6-, 12- and 18-month points than those without delirium and determine if delirium is an independent predictor of poorer ADL, IADL and BADL.
Hare et al. (2008) [18]	28 elderly patients	Audit	Emergency Department (Australia)	Determine if routine cognitive screening of elderly patients in emergency department could lead to early identification of delirium.
Hare et al. (2014) [19]	320 older patients	Prospective observational study	Emergency Department (Australia)	Derive a brief screening tool to predict the presence of delirium
Kennedy et al. (2014) [20]	700 elderly patients	Prospective observational study	Urban tertiary care emergency department (United States)	Create a risk prediction rule for emergency department delirium and compare mortality rates and resource utilization of delirious versus non-delirious elderly patients
Singler et al. (2014) [21]	133 elderly patients	Prospective single center cohort study	Interdisciplinary emergency department of an university-affiliated hospital (Germany)	Assess the prevalence of delirium and its detection by emergency department physicians, and identify delirium-associated patient characteristics
Modified Confusion Assessment Method for the Emergency Department (mCAM-ED) (Grossmann et al. 2014) [22]				
Grossmann et al. (2014) [22]	207 elderly patients	Prospective observational study	Emergency Department of the University Hospital Basel, (Switzerland)	Investigate whether there is a need for a standardized delirium screening and assessment instrument in the ED; evaluate the feasibility of a new algorithm for delirium screening, detection and managementof delirium in the ED; assess interraterreliability of the developed mCAM-ED.
Confusion Assessment Method for the Intensive Care Unit (CAM-ICU) (Ely et al. 2001) [23]				
Han et al. (2009) [24]	341elderly patients	Prospective cross-sectional study	Tertiary care academic emergency department (United States)	Determine whether nursing home patients are more likely than non-nursing home patients to present to the emergency department with delirium and to explore how variations in their delirium risk facto profiles contribute to this association.
Han et al. (2009) [24]	303 elderly patients	Prospective cross-sectional study	Tertiary care, academic emergency department (United States)	Determine how often delirium is missed in emergency department; identify delirium risk factors in older emergency department patients; to characterizes delirium by psychomotor subtypes in the emergency department setting.
Han et al. (2010) [4]	629 elderly patients	Prospective cohort study	Tertiary care, academic emergency department (United States)	Determine if delirium is an independent predictor of 6-month mortality and assess if this relationship is modified by nursing home residence.
Han et al. (2011) [25]	202 elderly patients	Cross-sectional study	Tertiary care, academic emergency department (United States)	Determine how delirium and dementia affect the accuracy of the presenting disease and discharge instruction comprehension in older emergency department
Han et al. (2011) [26]	628 elderly patients	Prospective cohort study	Tertiary care, academic emergency department (United States)	Determine if delirium in the emergency department was an independent predictor of prolonged hospital length of stay
Suffoletto et al. (2013) [27]	259 elderly patients	Prospective study	Teaching hospital emergency departments (United States)	Study whether emergency physicians identify delirium and examine each of the four individual features of delirium separately to determine the variation in identification across features
Mariz et al. (2013) [5]	283 adult patients	Prospective cohort study	Urban tertiary care hospital (Portugal)	To determine delirium prevalence in an EDIMCU and assess routine biochemical parameters that might influence delirium occurrence
Han et al. (2014) [7]	406 elderly patients	Prospective observational study	Tertiary care, academic emergency department (United States)	Determine CAM-ICU validity and reliability in older emergency department patients
Sri-on et al. (2015) [28]	232 elderly patients	Prospective cross-sectional study	Emergency department of an urban tertiary care hospital (Thailand)	Determine the prevalence of delirium and identify risk factors and short-term outcomes in delirious elderly emergency department patients.
Hsieh et al. (2015) [29]	260 elderly patients	Prospective cohort study	Urban tertiary care hospital (United States)	Measure the prevalence and incidence of delirium in older adults as they transition from the emergency department to the inpatient ward; determine the association between delirium during early hospitalization and subsequent clinical deterioration
Delirium Triage Screen (DTS) (Han et al. 2013) [9]				
Han et al. (2013) [30]	406 elderly patients	Prospective observational study	Tertiary care, academic emergency department (United States)	Determine the diagnostic performances of novel assessments using the psychiatrist’s assessment as the reference standard
Brief Confusion Assessment Method (bCAM) (Han et al. 2013) [30]				
Han et al. (2013) [30]	406 elderly patients	Prospective observational study	Tertiary care, academic emergency department (United States)	Determine the diagnostic performances of novel assessments using the psychiatrist’s evaluation as the reference standard
Rizzi et al. (2015) [31]	239 elderly patients	Observational, prospective, multicentric and cross-sectional study	Emergency department (Spain)	Investigate the presence of delirium at admission in patients with decompensated heart failure, identify their risk factors and analyze their impact on clinical outcomes
Neelon and Champagne Confusion Scale (NEECHAM) (Neelon, 1996) [32]				
Almató et al. (2012) [33]	90 elderly patients	Prospective observational study	Emergency monitoring area (Spain)	Estimate the prevalence of delirium in the emergency monitoring area and analyze the association between the presence of delirium with risk factors and precipitants.
Richmond Agitation-Sedation Scale (RASS) (Sessler et al. 2002) [34]				
Han et al. (2014) [35]	1084 non-comatose elderly patients	Prospective cohort study	Tertiary care, academic emergency department (United States)	Determine if impaired arousal at initial presentation in older acutely ill patients predicted 6-month mortality and if this relationship was present in the absence of delirium.
Han et al. (2015) [36]	406 elderly patients	Prospective observational study	Tertiary care, academic emergency department (United States)	Determine the diagnostic accuracy of the RASS for delirium

**Table 2:** Summary of the psychometric aspects of validated delirium screening instruments.

Name and Author	Validation Sample	Country of Validation	Number of Items/Criteria; Administration Time	Sensitivity; Specificity	Validation in Other Countries
CAM (Inouye et al. 1990) [8]	56 patients	USA	9 criteria; 20 min	94% to 100%; 90% to 95%	Brazil, Finland, France, Germany, Portugal, Spain, Thailand
mCAM-ED (Grossmann et al. 2014) [22]	207 elderly patients	Switzerland	3 step assessment; 4–6 min	Small sample not able to calculate with accuracy	NF
CAM-ICU (Han et al. 2014) [7]	406 elderly patients	USA	4 criteria; 2–5 min	68% to 72%; 98.6%	NF
DTS (Han et al. 2013) [9]	406 patients	USA	Two-step assessment; 20 s	98%; 55%	NF
bCAM (Han et al. 2013) [30]	406 patients	USA	4 criteria; 2 min	78% to 84%; 96% to 97%	NF
NEECHAM (Neelon, 1996) [32]	426 elderly patients	USA	9 items; 10 min	95%; 78%	Portugal; Sweden; Belgium
RASS (Sessler et al. 2002) [34]	293 ICU patients	USA	Ten-step assessment; 30–60 s	99%; 64%	Brazil; Germany; Iran; Spain

CAM = Confusion Assessment Method; CAM-ICU = Confusion Assessment Method for the Intensive Care Unit; mCAM-ED = Modified Confusion Assessment Method for the Emergency Department; BCAM = Brief Confusion Assessment Method; RASS = Richmond Agitation-Sedation Scale; DTS = Delirium Triage Scale; OBS = Organic Brain Syndrome Scale; NEECHAM = Neelon and Champagne Confusion Scale; NF = Not Found, unable to find any published peer-reviewed articles regarding instrument validations in other countries.
